# Neuroprotective and antiepileptogenic effects of combination of anti-inflammatory drugs in the immature brain

**DOI:** 10.1186/1742-2094-10-30

**Published:** 2013-02-26

**Authors:** Young Se Kwon, Eduardo Pineda, Stéphane Auvin, Don Shin, Andrey Mazarati, Raman Sankar

**Affiliations:** 1 Department of Pediatrics, Division of Neurology, David Geffen School of Medicine at UCLA, 22-474 MDCC in CHS, Los Angeles, CA 90095-1752, USA; 2 Department of Pediatrics, College of Medicine, Inha University, Incheon, Republic of Korea; 3 Department of Pediatric Neurology, Hôpital Robert Debré, INSERM U676, Paris, 75019, France; 4 Department of Neurology, David Geffen School of Medicine at UCLA, Los Angeles, CA 90095, USA

**Keywords:** Epilepsy, Anti-epileptogenesis, Hippocampus, Status epilepticus, Inflammation, IL-1β, COX-2

## Abstract

**Background:**

Inflammatory signaling elicited by prolonged seizures can be contributory to neuronal injury as well as adverse plasticity leading to the development of spontaneous recurrent seizures (epilepsy) and associated co-morbidities. In this study, developing rat pups were subjected to lithium-pilocarpine status epilepticus (SE) at 2 and 3 weeks of age to study the effect of anti-inflammatory drugs (AID) on SE-induced hippocampal injury and the development of spontaneous seizures.

**Findings:**

We selected AIDs directed against interleukin-1 receptors (IL-1ra), a cyclooxygenase-2 (COX-2) inhibitor (CAY 10404), and an antagonist of microglia activation of caspase-1 (minocycline). Acute injury after SE was studied in the 2-week-old rats 24 h after SE. Development of recurrent spontaneous seizures was studied in 3-week-old rats subjected to SE 4 months after the initial insult.

None of those AIDs were effective in attenuating CA1 injury in the 2-week-old pups or in limiting the development of spontaneous seizures in 3-week-old pups when administered individually. When empiric binary combinations of these drugs were tried, the combined targeting of IL-1r and COX-2 resulted in attenuation of acute CA1 injury, as determined 24 h after SE, in those animals. The same combination administered for 10 days following SE in 3-week-old rats, reduced the development of spontaneous recurrent seizures and limited the extent of mossy fiber sprouting.

**Conclusions:**

Deployment of an empirically designed ‘drug cocktail’ targeting multiple inflammatory signaling pathways for a limited duration after an initial insult like SE may provide a practical approach to neuroprotection and anti-epileptogenic therapy.

## Findings

Epilepsy affects approximately 1% of the population. The principal manifestations of the disease (seizures) as well as the associated co-morbidities exert a considerable toll on persons afflicted with this disorder. Despite treatment with anticonvulsant medications aimed at a number of pharmacological targets, approximately one-third of patients remain treatment-resistant [1]. Thus one of the most important benchmarks for epilepsy research agreed upon has been therapy to prevent the development of epilepsy, or anti-epileptogenesis [2].

At the present time no evidence-based treatment for the prevention of epilepsy and the associated co-morbidities exists. Clinical trials to address the prevention of post-traumatic epilepsy have mainly involved a number of anti-epileptic drugs (AED) and the results have been uniformly disappointing [3]. In the laboratory setting, a number of pharmacological and electrical methods can be employed to produce status epilepticus (SE), which produce hippocampal injury acutely, while spontaneous recurrent seizures (SRS) and neurocognitive and behavioral deficits develop as chronic sequelae. Treatment of experimental animals with AEDs chronically after a bout of SE has resulted in variable degrees of neuroprotection but has not produced discernible anti-epileptogenic effects [4-7]. A recent review summarizes data suggesting the potential for achieving anti-epileptogenesis by modulating inflammation after an initial insult such as SE [8]. A large body of data exists identifying a number of inflammation-associated mechanisms in mediating neuronal injury. We hypothesized that multiple pathways are activated after an insult, and that combination therapy leveraging more than one target may prove more efficacious in achieving neuroprotection and in modifying epileptogenesis.

Chronic post-SE animals with SRS (epileptic animals) demonstrate anatomical and electrophysiological evidence of a form of synaptic plasticity known as mossy fiber sprouting [9]. Mossy fibers are axons of the dentate granule cells which make synaptic contacts with the dendrites of the CA3 pyramidal cells and interneurons in the hilus which participate in feedback as well as feed-forward inhibition. In epileptic brains, which demonstrate loss of mossy fiber targets, these axons form recurrent connections to granule cell dendrites in the inner molecular layer of the dentate gyrus. This form of synaptic plasticity has been demonstrated in experimental models of limbic epilepsy as well as surgically resected hippocampi from humans as a treatment for medication-resistant temporal lobe epilepsy (TLE). The extent of sprouting does not appear to correlate with seizure density [10], while histological data suggest that the robustness of mossy fiber sprouting may reflect the extent of hippocampal injury [11].

Here, we report on the effect of an empirically derived combination therapy directed against inflammatory signaling pathways for a limited duration to achieve discernible neuroprotection, decrease in SRS, and mossy fiber sprouting in developing animals. Previous work in our laboratory established that 2-week-old rat pups respond with extensive CA1 injury with minimal accompanying hilar injury after SE induced by lithium-pilocarpine treatment [12]. Profound hilar injury is encountered in 3-week-old pups after SE and these animals are highly likely to develop SRS and their hippocampi demonstrate dense mossy fiber sprouting [12,13].

All experiments were performed in accordance with the policies of the National Institutes of Health. In order to study the effect of treatment acute on neuronal injury, we selected 2-week-old (postnatal day 14, P14) Wistar rat pups, which showed highly selective CA1 injury which was enhanced by inflammation induced with lipopolysaccharide (LPS) pretreatment [14]. Treatment with LPS enhanced kindling epileptogenesis at this age and animals followed for 3 months after lithium pilocarpine SE demonstrated more gliosis and a more severe epileptic phenotype [15]. In these experiments, rats were injected with lithium chloride (3 mEq/kg, i.p., given 16 to 18 h prior to s.c. injection of 60 mg/kg of pilocarpine) as described before [12-15]. In addition, these animals received 50 μg/kg of LPS i.p., immediately followed by i.p. injections of either vehicle (*n* = 6) or one of the following anti-inflammatory drugs (AID) minocycline (*n* = 5, 100 mg/kg, Sigma) because of its ability to inhibit microglial activation and attenuation of tumor necrosis factor signaling, as well as inhibiton of expression of caspase-1 and caspase-3, cyclooxygenase 2 inhibitor (COX-2 inhibitor) CAY10404 dissolved in DMSO (1 mg/kg, *n* = 6 or 10 mg/kg, *n* = 10) (Cayman Chemical, Ann Arbor, MI, USA), or recombinant interleukin-1 antagonist (rIL-1ra, 100 mg/kg, *n* = 5) (Amgen Inc., Thousand Oaks, CA, USA). We were the first to describe in detail the age-specific pattern of injury that is selective to the CA1 in P14 pups; the dentate hilus and the area CA3 are spared from SE-induced injury at this very young age [12]. The LPS injection was used to augment the selective CA1 injury in the P14 rat pup [14,15] such that even modest neuroprotection with our AID treatment regimens would be readily discernible. We also hypothesized that more than one inflammatory signaling pathway may participate in mediating acute injury as well as adverse plasticity leading to epileptogenesis. Thus, in a separate set of experiments, these P14 pups were injected with different binary combinations of these AIDs (rIL-1ra + COX-2 inhibitor (*n* = 5); rIL-1ra + minocycline (*n* = 5); COX-2 inhibitor + minocycline (*n* = 5)). Age-matched vehicle controls received DMSO or saline. All animals received diazepam 10 mg/kg i.p. 90 min after pilocarpine injection to improve survival. Histological analysis using hematoxylin and eosin was undertaken as described in our previous papers [12-15].

**Figure 1:**
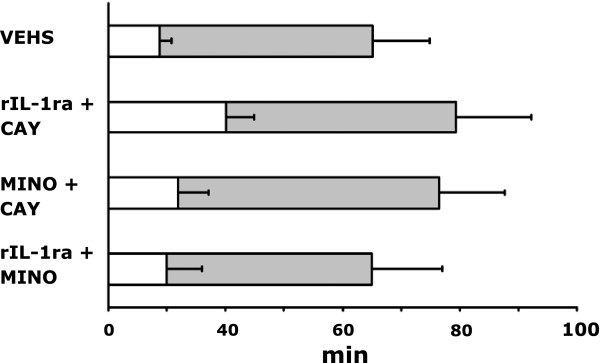
**Status epilepticus (SE) after administration of lipopolysaccharide (LPS) with or without anti-inflammatory drugs.** Mean (± SEM) of both time to onset (open bar) and total duration (open + filled bar) after pilocarpine treatment following a prior injection of LPS or LPS + combination of anti-inflammatory drugs. The latency to pilocarpine-induced seizure onset in combination treatment of COX-2 inhibitor plus rIL-1ra with LPS was significantly delayed as compared to those given vehicle (*P <0.01). There were no significant changes in total duration of SE among groups. CAY, COX-2 inhibitor; MINO, minocycline; rIL-1ra = recombinant interleukin-1 receptor antagonist; VEHS, vehicles.

**Figure 2:**
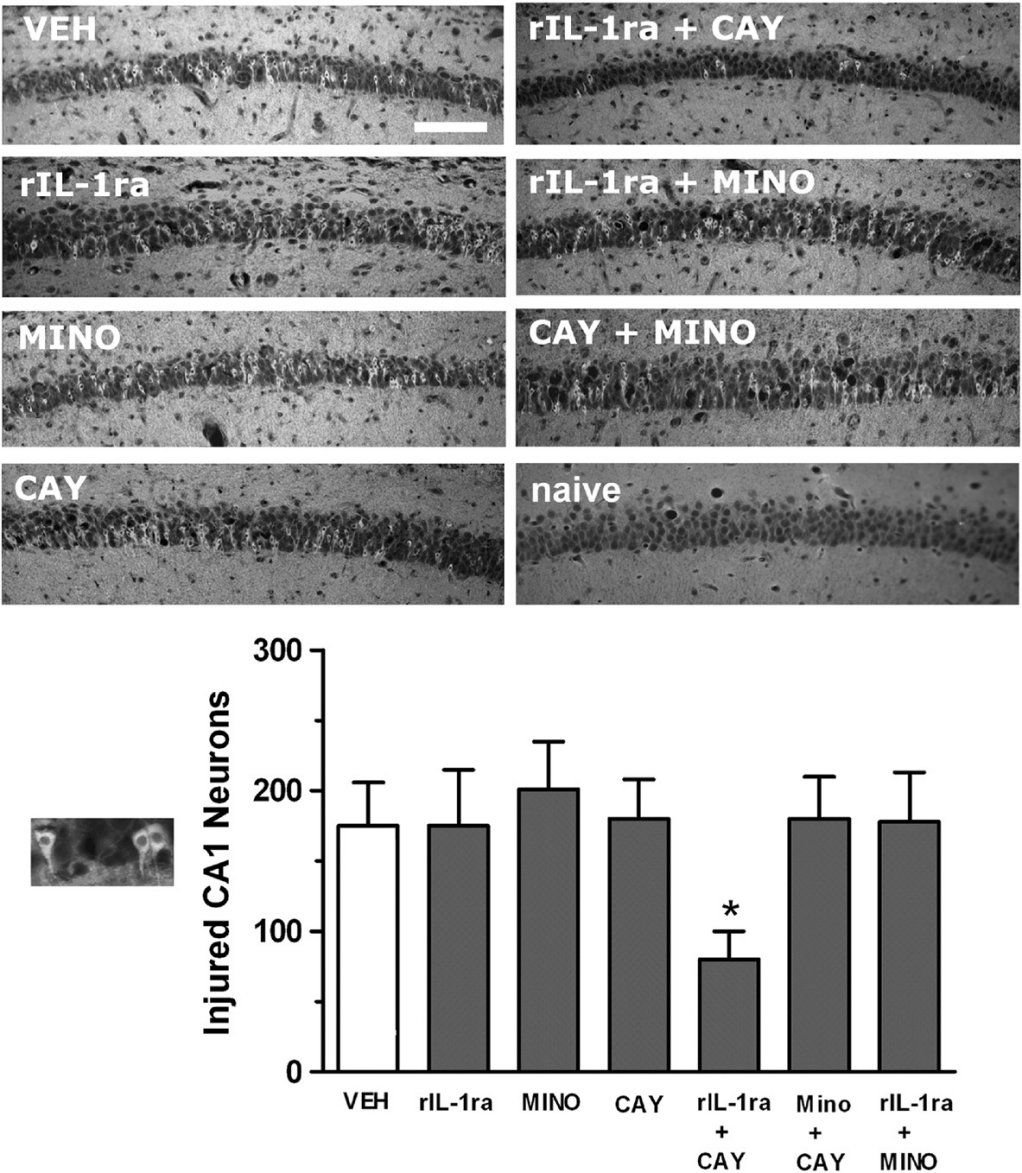
**Neuronal injury in hippocampal CA1 subfield after co-administration of anti-inflammatory drugs against LPS + pilocarpine-induced SE in 2-week-old rats.** Treatment with a single anti-inflammatory compound did not diminish the amount of CA1 hippocampal damage as compared to vehicle (left column). A combination treatment of rIL-1ra + CAY resulted in significant reduction of injured neurons which was not seen with any other combination (right column). Outset shows high magnification of eosinophilic neurons with pyknotic nuclei and irregular cell bodies that were counted as injured CA1 cells in the graph. Bars represent mean ± SEM. Quantification shows significant attenuation of neuronal injury resulted from combined administration of rIL-1ra + CAY administered prior to the onset of status (*P <0.05). CAY, COX-2 inhibitor (10 mg/kg); MINO, minocycline; naïve, age-matched normal rat (no seizures, no treatment); rIL-1ra, recombinant interleukin-1 receptor antagonist; VEH, vehicle. Scale bar is 50 μm.

Following systemic administration of pilocarpine, all rats showed typical seizure behaviors progressing to stage 3 or beyond. We found no differences in the latency to seizure onset in the presence of inflammation induced by LPS or when any of the anti-inflammatory drugs was used one at a time (data not shown). When binary combinations were tried, the combination rIL-1ra + COX-2 inhibitor (CAY 10404, 10 mg/kg) resulted in increased latency to seizure onset as compared to those given vehicle alone (40.1 ± 9.70 min *vs*. 17.5 ± 4.08, *P* <0.01) (Figure 1). However, the duration of seizure remained similar for all treatments. Figure 2 shows that only the combination of rIL-1ra + COX-2 inhibitor resulted in discernible neuroprotection of CA1 neurons as determined 24 h after SE.

Less than 30% of the rats subjected to SE at P14 develop SRS [12], and treatment with LPS increases that fraction to about 50% [15]. Our previous work [12] also showed that by P21, the hippocampal injury produced by lithium-pilocarpine SE extended beyond the CA1 region, involving also the hilar interneurons and CA3 neurons. Because >75% of the 3-week-old animals (P21) subjected to lithium pilocarpine SE developed SRS and dense mossy fiber sprouting in our previous studies [12,13], we deployed rats of this age to evaluate the efficacy of the rIL-1ra + COX-2 inhibitor combination in preventing epileptogenesis after lithium-pilocarpine SE. In this set of experiments animals were not primed with LPS because our goal was not to study injury and neuroprotection at 24 h, but to observe the rats in the long term for the development of epilepsy and to evaluate mossy fiber sprouting in the chronically epileptic animals. Previous work [12,13] has shown that at least 75% of P21 animals develop epilepsy 3 months or longer after lithium-pilocarpine SE.

Animals were treated with vehicle or one dose of the combination anti-inflammatory therapy immediately prior to the administration of pilocarpine. This sequence of administration was undertaken to ensure that the agents used in intervention are available even as the inflammatory cascade is being set into motion by the SE, such that a proof of principle as to the validity of the chosen targets can be established. That can set the background to explore in the future the window of opportunity for effective intervention after seizures have started. The only combination used was that involving rIL-1ra and the COX-2 inhibitor since none of the other regimens had resulted in discernible neuroprotection in the earlier set of experiments. A separate group of animals continued to receive once daily treatment with the anti-inflammatory cocktail for 10 days following SE. Four months after SE, animals were implanted with epidural electrodes as described in our other reports and subjected to a continuous EEG and video monitoring for a period of 3 weeks for the purpose of acquisition and analysis of spontaneous recurrent seizures. At the end of monitoring, animals were euthanized and brains were processed for the analysis of mossy fiber sprouting using Timm staining [12,13] and employing a 0–5 scale as previously described [16].

**Figure 3:**
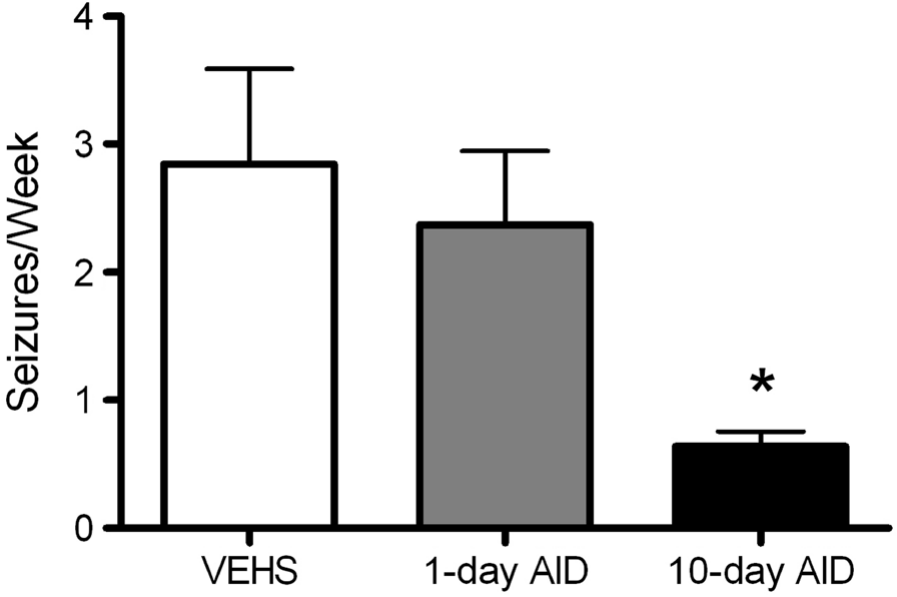
**Protracted anti-inflammatory drug treatment reduced the frequency of spontaneous recurrent seizures (SRS) among epileptic animals.** Bar graphs represent the average number of seizures observed. Over 3 weeks of continuous video and EEG monitoring, vehicle-treated animals (VEHS) presented an average of 2.9 ± 0.6 seizures per week, while those treated with a single injection of anti-inflammatory drug (AID) did not show significant reduction in seizure frequency and exhibited an average of 2.5 ± 0.4 seizures per week (*P* >0.5). Animals treated with daily injections of AID for 10 consecutive days showed a marked decrease in SRS frequency (*P <0.05 10 days of drug treatment *vs*. two other groups, *n* = 8).

Monitored at 4 months after SE at the age of 3 weeks, a single AID treatment consisting of IL-1ra and CAY did not result in a significant change in the number of rats developing SRS. Spontaneous seizures were observed in six of nine vehicle-treated rats, seven of nine rats with single drug injection, and in six of nine animals with 10-day treatment regimen. However, protracted (10-day) AID cocktail treatment did reduce seizure frequency among those animals with spontaneous seizures (Figure 3). Over 3 weeks of continuous video and EEG monitoring, vehicle-treated rats exhibited minimal-maximal-median seizures of 1-18-3; those which received a single injection: 2-13-3; and animals with 10 once-a-day injections: 2-4-2 (*P <0.05 10 days of drug treatment *vs*. one AID cocktail treatment or saline treatment).

**Figure 4:**
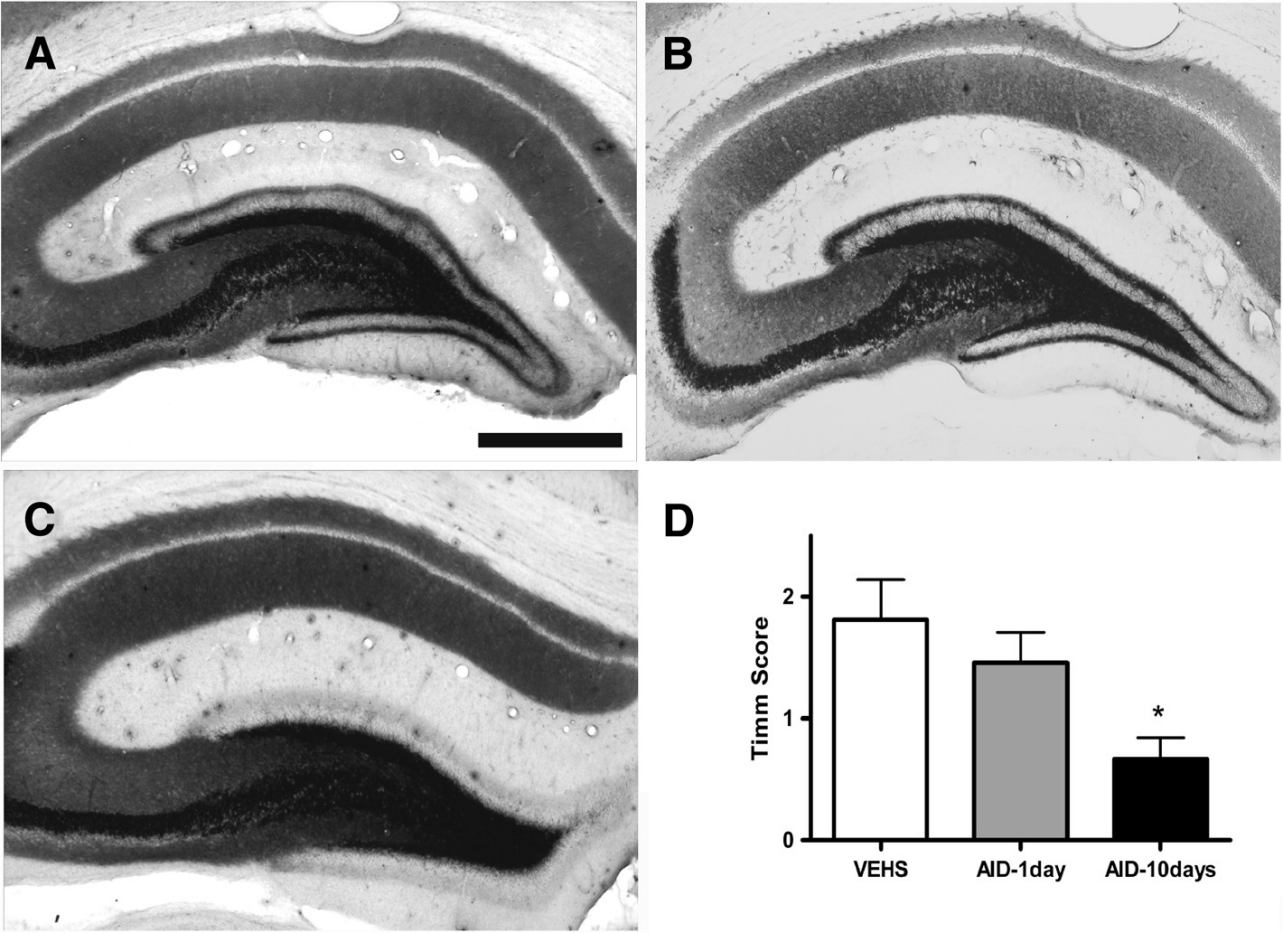
**Timm staining showing mossy fiber sprouting in supragranular region of the dentate gyrus of saline-treated and AID-treated animals.** (**A**) Vehicle treated. (**B**) The second group of animals was injected with AID for 1 day. (**C**) The third group received AID treatment for 10 consecutive days after induction of status epilepticus. Four months after SE, brains were harvested and processed for Timm staining. (**D**) Protracted AID (10-days) reduced mossy fiber sprouting as observed using Timm staining. Analysis of mossy fiber sprouting showed synaptic reorganization in vehicle-treated rats as well as in animals treated with a single injection of anti-inflammatory drugs (Timm scores 1.81 ± 0.32 and 1.46 ± 0.25, respectively). There was a statistically significant decrease in mossy fiber sprouting in the animals treated with anti-inflammatory drugs for 10 days (Timm score 0.67 ± 0.17); (*P* <0.05 *vs*. each of two other groups). Scale bar, 100 um.

The analysis of mossy fiber sprouting showed moderate synaptic reorganization in vehicle-treated rats as well as in animals treated with a single injection of anti-inflammatory drugs (Figure 4, Timm scores, mean scores ± SEM: 1.81 ± 0.32 and 1.46 ± 0.25, respectively). There was a statistically significant decrease in mossy fiber sprouting in the animals treated with anti-inflammatory drugs for 10 days (Timm score 0.67 ± 0.17; *P* <0.05 *vs*. one AID cocktail treatment or saline treatment).

The disappointing results with sustained *AED* treatment of post-SE animals to prevent epileptogenesis have directed research into other molecular targets. Rather than modifying neuronal excitability via influencing ion channel conductance, interest has turned to addressing pathways that may have a greater effect on plasticity resulting from seizures. Of those, the neuroinflammation-related pathways seem to be receiving attention of late [8]. Cyclooxygenase-2 inhibitor celecoxib has been reported to have a beneficial effect on epileptogenesis following lithium pilocarpine SE in both mature [17] and developing [18] rats. However, other investigators found parecoxib, another COX-2 inhibitor, to be neuroprotective but not antiepileptogenic [19] in the pilocarpine model of TLE. However, Holtman *et al*. [20,21] found that not only was the COX-2 inhibitor SC-58236 ineffective as an anti-epileptogenic agent [20] in a rat model of epilepsy after electrically-induced SE, it actually produced seizure deterioration and increased mortality [21]. Number of differences may account for the discrepancy between the results of Holtman *et al*. [20,21] and those of ours and other investigators [17-19]. The model of SE employed in the Holtman *et al*. [20,21] studies involved induction of SE electrical stimulation of the hippocampus rather than treatment with lithium-pilocarpine. The SE was much longer in duration (up to 9 h compared to 60 to 90 min in the pilocarpine studies, in which the animals received a diazepam dose). It is not possible to speculate as to whether there was some unique toxicity to the specific COX-2 inhibitor used in that study. Consistent with the expected safety and possible benefit of COX-2 inhibition in the treatment of pilocarpine SE, mice with conditional ablation of the COX-2 gene in the forebrain enjoyed diminished mortality and some improvement in memory performance after pilocarpine-induced SE [22].

Blocking the synthesis of interleukin-1β biosynthesis by an interleukin converting enzyme antagonist has shown potential in the model of kindling epileptogenesis [23] as well as epilepsy induced by kainic acid treatment [24]. The availability of the human recombinant interleukin-1 receptor antagonist (rIL-1ra) anakinra prompted us to evaluate its anti-epileptogenic potential, especially since its transport across the blood–brain barrier [25,26] appears to be adequate for modifying IL-1β signaling in the brain. Our experiments showed it to be effective when combined with the COX-2 selective inhibitor, CAY 10404. Despite concerns about the potential for fibroblast growth factor-2 (FGF-2) to increase excitability [27], localized delivery of FGF-2 and brain-derived neurotrophic factor (BDNF) by employing viral vectors has demonstrated potential for anti-epileptogenesis [28,29].

In humans, brain injury from a number of causes such as SE, traumatic brain injury, hypoxic-ischemic encephalopathy, stroke, and so on, give rise to variable incidences of epilepsy after varying latencies. The classic post-traumatic epilepsy prevention studies [3] subjected patients to prolonged treatment with AEDs with significant toxicities and side effects, and still failed to prevent the development of epilepsy. Our results highlight that both limited neuroprotection and a modicum of anti-epiletogenic disease modification can be achieved by targeting inflammation. However, the translationally important message is that while evidence exists supporting the role of many individual inflammatory pathways, only a specific combination therapy provided discernible benefits. Further, the AID cocktail treatment protocol involved a limited duration, unlike the chronic AED regimens that have been tried in humans as well as animal models. The importance of our findings for clinical translation are that our intuition-driven empiric AID cocktail design leverages elucidated mechanisms involving specific pathways while enabling: (1) treatment with drug classes that have already been evaluated for safety and approved for human use; (2) short duration treatment with drug classes that are already in use for chronic conditions; and (3) does not involve introduction of viral vectors into the CNS. It remains to be shown if the doses and duration of the regimen can be optimized for greater efficacy, and if such treatment may also modify the evolution of epilepsy-associated co-morbidities that seem to impact on the quality of life of patients even more than seizure frequency.

## Competing interests

None of the authors has any conflicts of interest with any commercial entities.

## Authors’ contributions

YSK, EP, SA, and DS performed all the experiments, data analysis, and also collaborated in writing. AM assisted with study design and data analysis. RS participated in the design of the studies, data analysis, and finalization of the manuscript. All authors have read and approved the final version of the manuscript.
